# Parental bonding and neuropsychological performance are associated with episodic simulation of future events in trauma‐exposed patients with major depressive disorder

**DOI:** 10.1002/brb3.474

**Published:** 2016-05-08

**Authors:** Melissa Parlar, Alex Lee, Zeeshan Haqqee, Latisha Rhooms, Ruth A. Lanius, Margaret C. McKinnon

**Affiliations:** ^1^ McMaster Integrative Neuroscience Discovery and StudyMcMaster UniversityHamiltonOntarioCanada; ^2^ Mood Disorders ProgramSt. Joseph's HealthcareHamiltonOntarioCanada; ^3^ Department of PsychiatryUniversity of Western OntarioLondonOntarioCanada; ^4^ Department of Psychiatry and Behavioural NeurosciencesMcMaster UniversityHamiltonOntarioCanada; ^5^ Homewood Research InstituteGuelphOntarioCanada

**Keywords:** Autobiographical memory, childhood attachment, depression, episodic simulation, neuropsychology, relationship quality, trauma

## Abstract

**Introduction:**

Major depressive disorder (MDD) and trauma‐related disorders are associated with deficits in remembering the past and imagining the future (i.e., episodic simulation). We examined parental bonding and neuropsychological performance in relation to episodic simulation in trauma‐exposed patients with recurrent MDD.

**Methods:**

Trauma‐exposed patients with MDD (*n* = 21) and matched controls (*n* = 20) completed a future‐oriented Autobiographical Interview, the Parental Bonding Instrument, and a standardized neuropsychological battery.

**Results:**

Patients with major depressive disorder generated fewer episodic details for future neutral events compared to controls. Although higher reported levels of maternal care were associated with increased specificity of negative future events among the patient group, higher maternal overprotection was related to decreased specificity of negative and positive future events. Higher levels of performance on measures of intelligence, verbal memory, executive functioning, and sustained attention were associated with increased specificity of future events.

**Conclusions:**

Maternal relations during childhood and neuropsychological performance are related to the specificity of episodic simulation in adult patients with MDD. Childhood experience continues to influence memory performance into adulthood.

## Introduction

A substantial body of evidence suggests that patients with major depressive disorder (MDD) (King et al. 2010) and with posttraumatic stress disorder (PTSD) (Moore and Zoellner 2007) are impaired in the retrieval of autobiographical memories (AM). In particular, these populations show a bias toward overgeneralized memory recall, where retrieval consists primarily of recollection of details related to repeated or long‐standing events (non‐episodic or semantic details), as opposed to the heightened recall of details specific in time and place (episodic details) seen in healthy populations. Episodic simulation is the construction of future events, achieved through drawing on episodic memories of past experiences and recombining and elaborating upon them, and is thought to require our awareness of subjective time (autonoetic awareness) (Tulving 2002; Schacter and Addis 2007). The ability to imagine future life events, or, episodic simulation, relies on many of the same cognitive, psychological, and neural processes known to be involved in AM (Spreng and Levine 2006; Schacter and Addis 2007; Hach et al. 2014). Although impairments in AM and in episodic simulation are well established in patients with depression and PTSD, factors contributing to the development of these deficits remain poorly understood. Here, we explore the relation between parental attachment, thought central to the emergence of autobiographical memory in childhood (Harley and Reese 1999; Nelson and Fivush 2004; Fivush et al. 2006), neuropsychological performance, and the ability to imagine future events in patients with a history of recurrent MDD and trauma exposure, primarily developmental in nature, and likely to have involved alterations in childhood attachments.

A number of studies reveal alterations in episodic simulation of future events in patients with MDD (MacLeod and Byrne 1997; Holmes et al. 2008; Bjärehed et al. 2010; King et al. 2011a; Morina et al. 2011; Kosnes et al. 2013) and with PTSD (Brown et al. 2013; Kleim et al. 2014) with some studies pointing towards a moderating influence of emotion on episodic simulation. For example, Kleim et al. (2014) found that assault and motor vehicle accident survivors with PTSD imagined fewer specific future events in response to positive cues, as compared to trauma‐exposed participants without PTSD. Brown et al. (2013), however, found that combat veterans with PTSD were more likely to generate overgeneral future events in response to neutral cue words as compared to combat veterans without PTSD. In addition, patients with depression have repeatedly shown impairments in the generation of future positive events (Bjärehed et al. 2010; Morina et al. 2011; Kosnes et al. 2013). Preliminary work indicates that clinical variables, including burden of illness and impaired social functioning, are associated with reduced generation of future episodic details among patients with mood disorders (King et al. 2011a,b). The impact of parental bonding on episodic simulation has not yet been studied, despite knowledge that many patients with MDD suffer adverse childhood experiences likely to impact parental attachments. Indeed, in a sample of two thousand participants with anxiety and/or depressive disorders, only 8.8% *failed* to report experiencing a potentially traumatic or bothersome life event (Spinhoven et al. 2014). These experiences are likely to impact significantly on autobiographical recall. In one study, the left hippocampus, a region central to AM, of patients with MDD and adverse experiences was 18% smaller in volume than that of MDD patients without adverse experiences, likely due to enhanced HPA‐axis dysregulation among the trauma‐exposed group (Joëls 2011).

Indeed, attachment orientations have been associated with individual differences in AM development and recollection (Harley and Reese 1999; Nelson and Fivush 2004; Fivush et al. 2006). In one influential theory, mothers who are elaborative in discourse with their children, providing rich background details and asking open‐ended questions, have children who showed more detailed AM than did children of nonelaborative mothers (e.g., mothers who provide less background to the discussion and ask less open‐ended and more repetitive questions) (Reese and Fivush 1993). This led Nelson and Fivush (2004) to posit that the level of detail and richness in parental discourse influences strongly the development of AM. This relation appears bidirectional, where the children of highly elaborative mothers also show more secure attachment (Fivush and Vasudeva 2002). More recent work focuses on the role of attachment in the processing of negative emotions and memories and points toward attachment‐related individual differences in how children encode, store, and recall negative memories (Chae et al. 2011). For example, in one study of undergraduate students, simply activating the mental representation of an attachment figure following the retrieval of a distressing autobiographical memory led to an enhanced regulation of negative affect in a sample of observed undergraduate students (Selcuk et al. 2012), suggesting that, even in adulthood, attachment figures play a role in the processing of emotional AM.

In addition to attachment orientations, cognitive functioning has also reliably been shown to relate to AM retrieval (for a review, see King et al. 2010). Deficits in working memory (Raes et al. 2005) verbal fluency (Dalgleish et al. 2007; Sumner et al. 2014), verbal memory (Spinhoven et al. 2006), and problem solving (Arie et al. 2008) are correlated with reduced specificity of AM retrieval in this population. Further, executive control affects search processes and monitoring of retrieval output of AM (Conway and Pleydell‐Pearce 2000). Indeed, patients with PTSD have shown elevated production of non‐episodic details during AM recall of both traumatic/negative and neutral events. This could reflect poor executive control and monitoring of extraneous information during event recall (McKinnon et al. 2014). Research examining the impact of cognitive functioning on episodic simulation in this population is scarce. Trauma exposure and MDD have, individually, been linked to disruptions in a highly similar range of fronto‐temporally mediated cognitive functions, including recollective memory (Yehuda et al. 2004; Talarowska et al. 2010), working memory (Vasterling et al. 2002; Gałecki et al. 2013), processing speed (McDermott and Ebmeier 2009; Cohen et al. 2013), and cognitive flexibility (Polak et al. 2012; Snyder 2012). Given the impairments in cognitive functioning observed among these populations and the overlapping cognitive processing resources required for both AM retrieval and imagining future events (Schacter and Addis 2007), it is important to assess the role of cognitive functioning in episodic simulation in this population.

This study aimed to examine the role of both childhood attachments and neuropsychological performance in episodic simulation of future events among trauma‐exposed patients with recurrent MDD. Participants were asked to imagine and describe future events in relation to positive, negative, and neutral cue words, allowing us to further examine the moderating role of emotional valence on episodic simulation. Details related to the imagined event were scored as internal (i.e., episodic and reflective of memory specificity), whereas details unrelated to the imagined event, or representing semantic knowledge or repeated events, were scored as external (i.e., non‐episodic and reflecting poor memory specificity) (Levine et al. 2002). We posited that parental attachments in childhood would continue to impact autobiographical episodic simulation in adulthood. Further, we predicted that poorer neuropsychological performance would be associated with reduced generation of episodic details in our patient group. In line with previous research, we also predicted that patients with MDD and comorbid trauma exposure would show overgeneral episodic simulation compared to controls. Finally, we examined latency to generate future events to determine if participants with MDD and trauma exposure were slower than controls at producing future events.

## Method

### Participants

This study was approved by the Hamilton Integrated Research Ethics Board of McMaster University and St. Joseph's Healthcare, Hamilton. Twenty‐one right‐handed patients (mean age: 41.3(14.5), 10 males, 11 females) who met DSM‐IV diagnostic criteria for a primary diagnosis of recurrent (i.e., ≥3 episodes) MDD on the Structured Clinical Interview for DSM‐IV‐TR Axis I Disorders (SCID‐1; First et al. 1997) were recruited. All participants in the patient group had a history of trauma exposure, according to responses on the Clinician‐Administered PTSD Scale (CAPS; Blake et al., 1995) and/or Childhood Trauma Questionnaire (CTQ; Bernstein et al., 2003). Among patients with MDD, five met criteria for moderate‐to‐severe childhood abuse on the CTQ only, nine met criteria for lifetime PTSD or trauma exposure on the CAPS only, and seven met criteria for childhood trauma exposure on the CTQ and a diagnosis of PTSD or trauma exposure on the CAPS. Among those participants who met criteria for PTSD or trauma exposure on the CAPS, 11 experienced interpersonal trauma (e.g., abuse by caregiver) and five experienced single‐blow, accidental trauma (e.g., car accident). In total, six participants met criteria for current PTSD. A group of healthy controls (HC) consisted of 20 right‐handed sex‐, age‐ and education‐matched participants with no history of psychiatric illness or trauma exposure (mean age: 36.5 (13.4), 10 males, 10 females). Demographic and clinical characteristics of the study sample are summarized in Table 1.

**Table 1:** Clinical and demographic characteristics of study sample

Characteristic	MDD with trauma (*n* = 21)	Controls (*n* = 20)
	Mean (SD)	Mean (SD)
*Demographic Characteristics*		
Age	41.3 (14.5)	36.5 (13.4)
Years of education	15.2 (3.9)	16.9 (2.6)
Sex (female:male)	11:10	10:10
Ethnicity (Caucasian) *n* (%)	19 (90%)	17 (85%)
Employment status *n* (%)		
Employed	7 (33%)	17 (85%)
Unemployed	12 (57%)	0 (0%)
Student	2 (9%)	2 (10%)
Retired	0 (0%)	1 (5%)
*Clinical Characteristics*		
HAM‐D	*11.8 (6.1)	0.5 (0.9)
CAPS (month)	*29.2 (34.4)	0.0 (0.0)
Childhood Trauma Questionnaire		
Emotional Abuse	*12.9 (6.4)	7.5 (3.9)
Physical Abuse	7.0 (2.2)	5.9 (2.0)
Sexual Abuse	*7.7 (5.6)	5.0 (0.0)
Emotional Neglect	*13.7 (5.6)	8.5 (3.1)
Physical Neglect	*9.3 (5.3)	5.9 (1.3)
Number of depressive episodes	14.1 (15.2)	0 (0.0)
*Parental Bonding Characteristics*		
Parental Bonding Instrument		
Maternal Care	*20.7 (9.8)	29.5 (6.0)
Maternal Overprotection	14.6 (8.9)	12.6 (7.2)
Paternal Care	*19.0 (9.1)	27.3 (7.2)
Paternal Overprotection	9.7 (4.2)	10.4 (7.2)

CAPS, Clinician‐Administered PTSD Scale; HAM‐D, Hamilton Depression Rating Scale; MDD, major depressive disorder.

*P <* *0.05.

Participants were recruited from St. Joseph's Healthcare Hamilton. Those with a past or current diagnosis of bipolar disorder, a psychotic disorder, neurological disease, traumatic brain injury and/or head injury with loss of consciousness (lasting more than 60 sec), substance abuse in the last 6 months, current or lifetime history of substance dependence, and/or current or prior history of untreated significant medical illness were excluded. Participants were instructed not to use benzodiazepines within 12 h prior to testing. Patients were on a variety of medications, including selective serotonin reuptake inhibitors (*n* = 7), tricyclic antidepressants (*n* = 1), serotonin–norepinephrine reuptake inhibitors (*n* = 7), tetracyclic antidepressants (*n* = 2), noradrenergic and specific serotonergic antidepressants (*n* = 1), benzodiazepines (*n* = 5), bupropion (*n* = 5), second‐generation antipsychotics (*n* = 5), hypnotics (*n* = 1), anticonvulsants (*n* = 1), and no medications (*n* = 4).

### Measures

#### Future‐oriented autobiographical interview

A modified version of Crovitz's cue‐word test was used (Addis et al. 2009a; Baddeley and Wilson 1986). Participants were presented with three positive (i.e., friendly, pleasure, achievement), three negative (i.e., lie, afraid, broken), and three neutral (i.e., hat, tree, quiet) randomly ordered cue words, one at a time. The cue words were chosen from the ANEW database (Bradley and Lang 1999) and matched for concreteness, imagery, frequency, and meaningfulness (Paivio et al. 1968). After presentation of the cue word, participants were asked to orally describe in as much detail as possible a novel event related to that word, specific in time and place (i.e., autobiographical event) that could occur at some time in their future. Reaction time (RT) (i.e., latency to generate a future event in response to each cue word) was measured in seconds.

Following Addis et al. (2009a), if the participant could not think of an event in response to the cue word or if details were sparse, he or she was prompted no more than two times with cues from a standardized script that included phrases such as “What else may happen on that day?” or “Can you tell me a little bit more about that?”. Event descriptions were audio recorded for transcription and scoring.

Participants' imagined events were placed separately in a common pool and scored at random by three experienced raters who had achieved high interrater reliability and who were blind to group. The future event description for each word was segmented as either “internal” (i.e., episodic) or “external” (i.e., non‐episodic or semantic information) details (Levine et al. 2002). An example of an internal detail is “It was raining lightly when we descended into de Gaulle”. An example of an external detail is “Paris is the capital of France”. These details were summed to form internal and external composites for positive, negative, and neutral events.

#### Clinical assessments

Severity of depressive symptoms over the past week was assessed using the 17‐item Hamilton Rating Scale for Depression HAM‐D; Hamilton 1960). Current (i.e., past month) and past PTSD diagnostic status, symptom severity, and history of trauma exposure were assessed with the CAPS. The CTQ was administered to assess for severity of childhood: (1) emotional abuse, (2) physical abuse, (3) sexual abuse, (4) emotional neglect, (5) physical neglect. Participants also completed the Parental Bonding Instrument (PBI; Parker et al., 1979). The PBI is a 25‐item self‐report questionnaire designed to assess parental bonding through two perceived parenting styles of the mother and father during the first 16 years of life: (1) care (e.g., *my mother/father was affectionate to me*) and (2) overprotection (e.g., *my mother/father tried to control everything I did*). High care and low overprotection are considered optimal, whereas low care and high overprotection are considered least optimal. Each item is scored on a 4‐point scale ranging from 1 (very like) to 4 (very unlike) and assessed separately for mother and father. Scores on the PBI do not simply reflect current depressed mood state (Parker et al. 1979) and show good concordance with sibling ratings (Gotlib et al. 1988; Duggan et al. 1998).

#### Neuropsychological test battery

Fronto‐temporally mediated cognitive functioning was assessed using the following standardized neuropsychological measures. *Declarative memory*: (1) California Verbal Learning Test II (standard form) (CVLT‐II; Delis et al. 2000): a word learning task that provides measures of both immediate and delayed recall, and yes/no recognition. *Executive functioning*: (1) Color Trails Test (Parts 1 and 2) (D'Elia et al. 1996): measures attention, psychomotor speed (Part 1 and 2), and mental flexibility (Part 2); (2) Wisconsin Card Sorting Test (128‐item version) (WCST; Heaton 2003): a computerized task assessing one's ability to form and switch concepts based on feedback. *Attention*: (1) Conners' Continuous Performance Test – Second Edition (CPT‐II; Conners 2000): a computerized task assessing response inhibition and sustained attention. *Current intellectual functioning*: (1) Wechsler Abbreviated Scale of Intelligence (WASI; Wechsler 1999): the Matrix Reasoning (performance index) and Vocabulary (verbal index) subtests were administered. Consistent with previous studies examining future thinking (Bjärehed et al. 2010; Kosnes et al. 2013), a verbal fluency task assessing phonemic fluency (stimuli: F, A, S), the Controlled Oral Word Association Test, was administered to assess for group differences in verbal fluency abilities (Gladsjo et al. 1999).

### Statistical analysis

To examine group differences on the demographic, clinical, and neuropsychological variables, independent samples *t*‐tests or Mann–Whitney *U* tests were conducted, depending on normality of data (assessed with the Shapiro–Wilk test). Associations between scores on the future‐oriented autobiographical interview and clinical (i.e., HAM‐D, CAPS, CTQ subscale scores, number of depressive episodes), parental bonding, and cognitive variables were calculated using Spearman's *ρ* (two‐tailed).

Due to nonnormality of the scores on the future‐oriented autobiographical interview (Shapiro–Wilk, *P *<* *0.05), these scores were log transformed in order to perform a parametric mixed‐design analysis of variance. The log transformation, however, did not result in a normal distribution among all of the scores (Shapiro–Wilk, *P *<* *0.05). The scores on this measure were therefore analyzed using a mixed‐effects model for nonparametric data (Noguchi et al. 2012) (this method is robust for small sample sizes and outliers). Follow‐up group comparisons of performance on the future‐oriented autobiographical interview were conducted with nonparametric Mann–Whitney *U* tests (using non‐log transformed data). Group differences on the log‐transformed RT data were analyzed using independent samples *t*‐tests. Significance was set at *α* = 0.05 for all analyses. Analyses were conducted with SPSS 21 (IBM, Armonk, NY) and R 3.0 statistical software (R Foundation for Statistical Computing, Vienna, Austria).

## Results

### Demographic and clinical characteristics

The patient group reported significantly higher symptom severity on all clinical measures (i.e., HAM‐D, CAPS, CTQ) (see Table 1). Age, years of education, sex distribution, and verbal fluency performance did not differ significantly between the two groups. Participants with depression and trauma exposure reported significantly lower levels of maternal and paternal care as compared to HC (*U *=* *91.5, *z *=* *−2.93, *P *<* *0.001, *r *=* *−0.46; *U *=* *81.5, *z *=* *−2.88, *P *<* *0.001, *r *=* *−0.47, respectively). The reported levels of maternal and paternal overprotection did not differ between groups. Neuropsychological performance scores are reported in Table 2. The patient group performed significantly worse than the control group on the CVLT‐II long‐delay free recall and recognition conditions, the Color Trails Test Part 2, and the Matrix Reasoning subtest of the WASI.

**Table 2:** Raw scores on measures of neuropsychological performance

Cognitive Variable	MDD with trauma (*n* = 21)	Healthy controls (*n* = 20)
	Raw score, mean (SD)	Raw score, mean (SD)
CVLT‐II		
Trials 1–5 total	51.9 (9.3)	56.5 (10.1)
Short‐delay free recall	11.1 (2.4)	12.9 (3.2)
Long‐delay free recall	*11.2 (2.6)	13.4 (2.7)
Recognition hits	**14.6 (1.5)	15.6 (0.8)
Color Trails Test		
Part 1 (sec)	31.9 (13.3)	28.0 (8.7)
Part 2 (sec)	*66.9 (21.1)	52.5 (14.1)
WCST		
Total errors	23.8 (18.5)	19.9 (20.2)
Perseverative errors	12.1 (11.8)	10.9 (10.8)
CPT‐II		
Hit reaction time (msec)	396.0 (52.0)	394.8 (59.0)
Omissions	4.2 (12.7)	3.1 (7.9)
Commissions	14.7 (8.1)	11.8 (5.3)
WASI		
Matrix reasoning	**26.2 (4.8)	30.1 (3.5)
Vocabulary	65.0 (9.1)	66.9 (7.4)
Two‐subtest IQ	113.5 (13.2)	119.4 (12.7)
COWAT		
Phonemic fluency	44.8 (12.3)	42.6 (13.1)

COWAT, Controlled Oral Word Association Test; CPT‐II, Conners' Continuous Performance Test‐II; CVLT‐II, California Verbal Learning Test‐II; WASI, Wechsler Abbreviated Scale of Intelligence; WCST, Wisconsin Card Sorting Test.

*P <* *0.05, **P <* *0.01.

### Correlations between future‐thinking scores and clinical variables

No correlations emerged between future‐thinking scores and HAM‐D, CAPS, the CTQ subscales, or number of depressive episodes.

### Correlations between future‐thinking scores and parental bonding scores

Among the group with MDD, higher maternal care was associated with generation of fewer non‐episodic (external) details for negative events (*r*_*s*_ = −0.45, *P *=* *0.04, 95% CI [−0.74, −0.03]), reflecting greater memory specificity (see Table 3). The opposite pattern was seen with the maternal overprotection subscale, where higher maternal overprotection was associated with an increased number of non‐episodic details for negative events (*r*_*s*_ = 0.52, *P *=* *0.02, 95% CI [0.11, 0.78]), reflecting poorer memory specificity. Higher maternal overprotection was also associated with an increased number of non‐episodic details for positive events (*r*_*s*_ = 0.48, *P *=* *0.03, 95% CI [0.60, 0.78]), again reflecting poorer memory specificity. Paternal care and overprotection were not correlated with future‐thinking scores.

**Table 3:** Correlates of future‐thinking scores among trauma‐exposed sample with MDD

	Positive Episodic	Positive Non‐Episodic	Negative Episodic	Negative Non‐Episodic	Neutral Episodic	Neutral Non‐Episodic
*Attachment Variable*
Parental Bonding Instrument						
Paternal care	+0.12	−0.15	−0.27	+0.17	−0.01	−0.21
Maternal care	+0.01	−0.43	+0.09	*−0.45	+0.08	−0.30
Paternal overprotection	−0.26	0.02	−0.31	+0.08	−0.31	+0.02
Maternal overprotection	−0.13	*0.48	−0.14	*+0.52	−0.18	+0.22
*Cognitive Variable*						
CVLT‐II						
Trials 1–5 total	*+0.47	−0.07	+0.41	+0.28	+0.30	+0.30
Short‐delay free recall	*+0.45	+0.04	+0.42	+0.28	+0.39	−0.28
Long‐delay free recall	+0.29	−0.15	+0.13	+0.20	+0.16	+0.28
Recognition hits	−0.10	0.12	0.19	0.20	−0.01	0.26
Color Trails Test						
Part 1 (seconds)	+0.24	+0.04	−0.19	−0.21	+0.08	+0.03
Part 2 (seconds)	+0.03	+0.11	−0.14	−0.01	−0.01	+0.13
WCST						
Total errors	−0.42	+0.12	*−0.56	−0.06	*−0.53	+0.03
Perseverative errors	*−0.49	+0.12	**−0.62	−0.10	**−0.62	−0.08
CPT‐II						
Hit reaction time (msec)	−0.25	−0.22	−0.29	−0.35	−0.17	−0.34
Omissions	−0.38	−0.14	*−0.53	−0.11	*−0.52	−0.43
Commissions	+0.22	0.34	+0.12	+0.33	+0.08	+0.35
WASI						
Matrix reasoning	+0.12	−0.32	+0.29	−0.27	+0.21	−0.18
Vocabulary	+0.35	0.08	**+0.66	+0.04	*+0.52	+0.11
Two‐subtest IQ	+0.17	−0.04	*+0.53	−0.10	+0.36	+0.01
COWAT						
Phonemic fluency	+0.03	+0.002	+0.17	+0.21	+0.29	+0.01

COWAT, Controlled Oral Word Association Test; CPT‐II, Conners' Continuous Performance Test‐II; CVLT‐II, California Verbal Learning Test‐II; WASI, Wechsler Abbreviated Scale of Intelligence; WCST, Wisconsin Card Sorting Test.

All correlation values are Spearman's correlation coefficients.

*P <* *0.05, **P <* *0.01.

### Correlations between future‐thinking scores and cognitive variables

Several cognitive correlates emerged in relation to future‐thinking scores among the group with MDD, particularly in the domains of intelligence, verbal memory, executive functioning, and sustained attention (see Table 3). Better performance on the WASI Vocabulary subscale raw scores (a measure of crystallized intellectual functioning) was associated with generation of more internal (episodic) details for negative and neutral events (*r*_*s*_ = 0.66, *P *<* *0.01, 95% CI [0.32, 0.85]; *r*_*s*_ = 0.52, *P *=* *0.02, 95% CI [0.11, 0.78], respectively), reflecting better memory specificity. Moreover, higher IQ based on the WASI two‐subtest full‐scale IQ was associated with generation of more episodic details for negative events (*r*_*s*_ = 0.53, *P *=* *0.02, 95% CI [0.12, 0.78]), reflecting better memory specificity. Better performance on an index measure of verbal memory, the CVLT‐II Total Raw scores subscale (*r*_*s*_ = 0.47, *P *=* *0.03, 95% CI [0.05, 0.75]) and the CVLT‐II short‐delay free recall subscale (*r*_*s*_ = 0.45, *P *=* *0.04, 95% CI [0.05, 0.75]) were related to increased production of episodic details for positive events, again reflecting better memory specificity. On a task of executive functioning, the WCST, number of total errors was associated with reduced generation of episodic details for both negative and neutral events (*r*_*s*_ = −0.56, *P *=* *0.01, 95% CI [−0.80, −0.17]; *r*_*s*_ = −0.53, *P *=* *0.02, 95% CI [−0.78, −0.12], respectively), reflecting poorer memory specificity. More perseverative errors on the WCST were also associated with reduced generation of episodic details for positive, negative, and neutral events (*r*_*s*_ = −0.49, *P *=* *0.03, 95% CI [−0.76, −0.10]; *r*_*s*_ = −0.62, *P *<* *0.01, 95% CI [−0.83, −0.25]; *r*_*s*_ = −0.62, *P *<* *0.01, 95% CI [−0.83, −0.26], respectively), reflecting reduced memory specificity. Finally, on a measure of sustained attention and impulsivity (i.e., the CPT‐II), a greater number of omission errors, reflecting inattention, was associated with reduced production of episodic details for negative and neutral events (*r*_*s*_ = −0.53, *P *=* *0.02, 95% CI [−0.78, −0.13]; *r*_*s*_ = −0.52, *P *=* *0.02, 95% CI [−0.78, −0.12], respectively), again reflecting reduced memory specificity. No significant correlations emerged between scores on the Color Trails Test or Controlled Oral Word Association Test fluency scores and number of details generated on the future‐oriented autobiographical interview.

### Group differences on future‐thinking task performance

**Figure 1:**
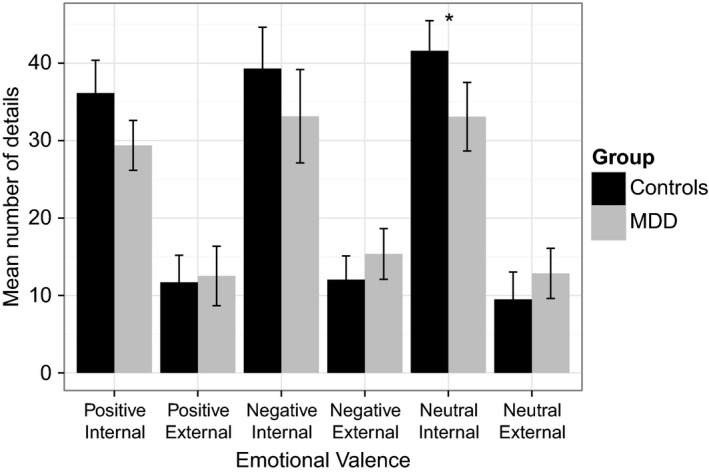
Mean number of internal (episodic) and external (non‐episodic) details generated by comparing healthy controls and trauma‐exposed patients with major depressive disorder on the future‐oriented autobiographical interview. Error bars indicated standard error of the mean. *P = 0.056.

On the future‐oriented autobiographical memory interview, there was a significant two‐way interaction between detail type (i.e., episodic or non‐episodic and group (i.e., MDD vs. HC) (*F*(1,39) = 4.0, *P *=* *0.04). Examining the mean number of episodic and non‐episodic details produced (log transformed) patients with MDD produced fewer episodic details than controls, however, this finding did not reach significance (*t* (37) = 1.5, *P *=* *0.14, *d *=* *0.48) (HC mean = 117.1, SD = 53.4; MDD mean = 95.6, SD = 58.1). There was a trending effect of valence, where patients with MDD produced fewer episodic details in response to neutral cue words as compared to controls (*U *=* *138, *z *=* *−1.88, *P *=* *0.056, *r *=* *−0.29) (HC mean = 41.6, SD = 17.3; MDD mean = 33.1, SD: 20.3) (see Fig. 1). Groups did not differ in their mean number of episodic details for positive cue words (*U *=* *167.5, *z *=* *−1.11, *P *>* *0.05, *r *=* *−0.17) (HC mean = 36.2, SD = 19.1; MDD mean = 29.4, SD = 14.8) or negative cue words (*U *=* *148.5, *z *=* *−1.39, *P *>* *0.05, *r *=* *−0.22) (HC mean = 39.3, SD = 23.9; MDD mean = 32.7, SD = 27.0). Groups also did not differ in the mean number of non‐episodic details for positive (*U *=* *195.0, *z *=* *−0.39, *P *>* *0.05, *r *=* *−0.06) (HC mean = 11.7, SD = 15.6; MDD mean = 12.5, SD = 17.6), negative (*U *=* *174.5, *z *=* *−0.69, *P *>* *0.05, *r *=* *−0.11) (HC mean = 12.1, SD = 13.7; MDD mean = 14.8, SD = 14.9), or neutral cue words (*U *=* *168.0, *z *=* *−1.10, *P *>* *0.05, *r *=* *−0.17) (HC mean = 9.5, SD = 15.7; MDD mean = 12.9, SD = 14.9).

Means for response latency to generate future events differed between groups for neutral cue words, where participants with MDD were significantly slower at generating a response compared to controls (*t* (37) = −2.1, *P *=* *0.04, *d *=* *−0.67) (HC mean = 35.2s, SD = 23.3; MDD mean = 71.0s, SD = 72.8). Groups did not differ in their latency in response to positive cue words (*t* (37) = −1.47, *P *>* *0.05, *d *=* *−0.47) (HC mean = 46.0s, SD = 34.8; MDD mean = 66.9s, SD = 57.3) or negative cue words (*t* (37) = −0.92, *P *>* *0.05, *d *=* *−0.30) (HC mean = 67.7s, SD = 45.7; MDD mean = 91.8s, SD = 57.3).

Exploratory analyses were performed, excluding the six participants with MDD who also met current criteria for PTSD. With these participants excluded from the analyses, groups no longer differed in their latency in response to neutral cue words (*t* (31) = −1.7, *P *=* *0.08, *d *=* *−0.58), suggesting that the participants with comorbid PTSD were driving the slower RT to neutral words. The remaining group comparison results remained consistent (i.e., nonsignificant).

## Discussion

To the best of our knowledge, this is the first study to examine the roles of parental bonding and neuropsychological performance in the specificity of episodic simulation in patients with a primary diagnosis of recurrent MDD and a history of trauma exposure, a population that reliably demonstrates deficits in AM recall and episodic simulation. The main finding in this study was that higher levels of maternal care were associated with reduced generation of non‐episodic details, reflecting increased specificity of episodic simulation in response to negative cue words and a greater ability to monitor retrieval output. Higher levels of maternal overprotection were related to increased production of extraneous details and poorer cognitive control operations in response to negative and positive cue words. In addition, higher levels of performance on neuropsychological measures of intelligence, verbal memory, executive functioning, and sustained attention were related to greater specificity of episodic simulation as indicated by a greater number of episodic details across positive, negative, and neutral memories. By contrast, levels of episodic simulation were unrelated to symptom severity, childhood trauma severity, and numbers of depressive episodes in this sample.

Higher levels of maternal care were associated with the generation of fewer non‐episodic details for negative cue words, reflecting increased specificity, whereas high levels of maternal overprotection were related to an increased production of non‐episodic details for negative and positive events, suggesting decreased specificity. These results suggest that attachment orientations, which are known to be involved in the development and retrieval of autobiographical memory even into adulthood, exert a protective effect on episodic simulation among individuals with depression that extends into adulthood (Harley and Reese 1999; Nelson and Fivush 2004; Selcuk et al. 2012). These results, in particular the correlations concerning details of negative future events, are in line with findings by Chae et al. (2011) who demonstrated that maternal attachment plays a role in the development of emotional AM, specifically for negative events. Experiencing higher levels of maternal care during childhood may contribute to the development of strategies that allow for more effective processing and elaboration of negative events well into adulthood (Chae et al. 2011). Notably, maternal care (and overprotection) was only associated with the production of external, non‐episodic details, suggesting that attachment plays a role in the increased (or decreased) production of details unrelated to the imagined event, rather than details specific in time and place concerning the future event. The reduced generation of extraneous details observed in association with maternal care likely reflects enhanced cognitive control and monitoring of mnemonic retrieval output, as impairments in cognitive control processes are related to elevated production of external/non‐episodic details, which were associated with maternal overprotection in this sample (Levine et al. 2002; McKinnon et al. 2008). There were no associations between levels of paternal bonding and episodic simulation. In one study, mother–child, but not father–child relationships, were predictive of the number of memories a young adult could retrieve from early life (Peterson and Nguyen 2010). Given the shared processes underlying AM retrieval and episodic simulation, our findings are in line with this report. Notably, Peterson and Nguyen (2010) found that father–child relationships predicted the age at which the earliest memory an adult could recall was encoded. The authors suggest that differences in how mothers and fathers engage in memory talk with children may provide a partial explanation of these findings.

Post‐traumatic stress disorder has been associated with elevated production of non‐episodic details during AM retrieval (McKinnon et al. 2014), likely related to reduced executive functioning. Indeed, future studies may examine whether the current finding that parental bonding is associated with production of non‐episodic details is mediated by executive control. Increased rumination or functional avoidance during response generation could also lead the participant to focus on and generate details unrelated to the event in question (i.e., non‐episodic details) as suggested by the CaR‐FA‐X model of overgeneral autobiographical memory that focuses on the impact of capture and rumination, functional avoidance, and impaired executive control in overgeneralized recall in participants with MDD (Williams et al. 2007).

Consistent with findings by King et al. (2011a), our results suggest that patients show a deficit in generation of episodic details related to imagining future events, whereas the absolute level of non‐episodic details generated did not differ between groups. Here, patients produced fewer internal details in response to neutral cue words than controls (results approached significance), and they were significantly slower than controls at generating responses for neutral events. A substantial literature indicates that negative and positive arousing events are better remembered than neutral events (Kensinger 2009). Based on our findings, this bias toward more easily remembering emotional events may extend to imagining future events. Indeed, compared to controls, patients appeared to show a selective deficit for neutral events. These results, however, conflict with previous studies examining episodic simulation in patients with MDD, which have shown selective deficits in the generation of future positive events (MacLeod and Byrne 1997; Holmes et al. 2008; Bjärehed et al. 2010; Morina et al. 2011; Kosnes et al. 2013). Methodological differences may account for these conflicting findings. Previous studies did not distinguish between episodic and non‐episodic details generated during the simulation of future events. Instead, task scores were based on overall vividness of imagined events or the total number of separate events imagined. Further, our patient sample differed from those in previous studies, as no existing studies, to the best of our knowledge, included only trauma‐exposed participants with depression. Future studies will need to compare patients with and without trauma exposure to systematically assess if history of trauma may account for these conflicting findings.

A highly specific association was found between higher levels of neuropsychological performance and increased production of episodic details. Better performance on measures of intelligence (WASI vocabulary subtest and full‐scale IQ scores), verbal memory (CVLT‐II total scores), executive functioning (WCST total and perseverative errors), and sustained attention (CPT‐II omissions) was associated with increased specificity of future events (i.e., greater number of internal details generated). Here, we have demonstrated that the association between neuropsychological performance and future‐thinking scores was specific to internal details, suggesting that neuropsychological functioning promotes enhanced episodic recall. Indeed, deficits in verbal memory (Yehuda et al. 2004; Talarowska et al. 2010), executive functioning (Polak et al. 2012; Snyder 2012), and sustained attention (van der Meere et al. 2007) are reported in patients with MDD and trauma‐related disorders, and these processes are further thought to be involved in AM retrieval (Spinhoven et al. 2006; Arie et al. 2008), and by extension, episodic simulation of future events. Verbal memory was only associated with positive future events, whereas intelligence and sustained attention scores were related to negative and neutral events, and executive functioning scores were related to all three emotional valences. A variable related to cognitive functioning that is worth considering for future studies is dissociation. Dissociative symptoms have been shown to be present in patients with MDD, and tend to be more severe in those with a history of childhood trauma (Molina‐Serrano et al. 2008; Žikić et al. 2009). Dissociation is related to poorer neuropsychological functioning in patients with PTSD (Roca et al. 2006), borderline personality disorder (Haaland and Landrø 2009; Krause‐Utz et al. 2012), and also HC (Brewin and Mersaditabari 2013; Brewin et al. 2013). Future studies should examine if dissociative symptoms may be mediating the relation between neuropsychological performance and episodic simulation in trauma‐exposed patients with MDD.

There are several limitations to this study that require consideration, such as the small sample size and retrospective nature of the PBI. Notably, however, scores on the PBI have shown good concordance with sibling ratings (Gotlib et al. 1988; Duggan et al. 1998) and do not simply reflect a depressed mood state (Parker et al. 1979). Further, while the majority of our patient sample reported moderate‐to‐severe childhood trauma, the type of trauma exposure remained relatively heterogenous, as a subset of participants reported experiencing only single‐incident trauma. Future studies should systematically assess if specific subtypes of trauma differentially impact episodic simulation. It will also be important to explore clinical heterogeneity by examining the impact of comorbid personality disorders on performance. In this study, the majority of the patient sample were taking psychiatric medications at the time of testing; future studies may wish to control for the use of medications. Notably, the average age of our sample was approximately 41 years of age. Participants grew up in an era where the majority of child care was provided by mothers, perhaps explaining further the specificity of the relation between maternal (but not paternal) care and episodic memory performance. Finally, the mechanisms underlying parental bonding and future thinking remain unknown, and future studies should assess potential mediators of this association, such as increased rumination, functional avoidance, or emotional dysregulation during episodic simulation.

This study is the first to examine attachment and neuropsychological performance in relation to episodic simulation of future events, drawing on past theories proposing that maternal attachments and cognitive processes play crucial roles in the development and retrieval of AM. The findings of this study have important clinical implications. Interventions for trauma‐related disorders and depression aim to decrease patients’ negative biases toward the future and increase patients’ positive biases. Difficulties in imagining and rescripting future events may reduce one's ability to engage in this therapeutic process. Attachment with caregivers is an important developmental variable, as evoking stable attachment figures promotes emotional regulation during processing of negative personal memories (Selcuk et al. 2012) and is related to memory performance in adulthood. Attachment, together with neuropsychological dysfunction in patients with MDD and trauma‐related disorders, should be taken into account during treatment planning as they may ultimately have an impact on one's ability to engage successfully in therapy.

## Conflict of Interest

None declared.

## References

[brb3474-bib-0002] Addis, D. R. , D. C. Sacchetti , B. A. Ally , A. E. Budson , and D. L. Schacter . 2009a Episodic simulation of future events is impaired in mild Alzheimer's disease. Neuropsychologia 47:2660–2671. 19497331 10.1016/j.neuropsychologia.2009.05.018 PMC2734895

[brb3474-bib-0003] Arie, M. , A. Apter , I. Orbach , Y. Yefet , and G. Zalzman . 2008 Autobiographical memory, interpersonal problem solving, and suicidal behavior in adolescent inpatients. Compr. Psychiatry 49:22–29. 18063037 10.1016/j.comppsych.2007.07.004

[brb3474-bib-0004] Baddeley, A. , and Wilson, B. 1986 Amnesia, autobiographical memory and confabulation pp. 225–252 *In* Rubin D. C., ed. Autobiographical memory. Cambridge University Press, Cambridge, U.K.

[brb3474-bib-0005] Bernstein, D. P. , J. A. Stein , M. D. Newcomb , E. Walker , D. Pogge , T. Ahluvalia , et al. 2003 Development and validation of a brief screening version of the Childhood Trauma Questionnaire. Child Abuse Negl. 27:169–190. 12615092 10.1016/s0145-2134(02)00541-0

[brb3474-bib-0006] Bjärehed, J. , A. Sarkohi , and G. Andersson . 2010 Less positive or more negative? Future‐directed thinking in mild to moderate depression. Cogn. Behav. Ther. 39:37–45. 19714541 10.1080/16506070902966926

[brb3474-bib-0007] Blake, D. D. , F. W. Weathers , L. M. Nagy , D. G. Kaloupek , F. D. Gusman , D. S. Charney , et al. 1995 The development of a clinician‐administered PTSD scale. J. Trauma. Stress 8:75–90. 7712061 10.1007/BF02105408

[brb3474-bib-0008] Bradley, M. M. , and P. P. J. Lang . 1999 Affective Norms for English Words (ANEW): Instruction Manual and Affective Ratings. Psychology, Technical(C‐1), 0. http://doi.org/10.1109/MIC.2008.114

[brb3474-bib-0009] Brewin, C. R. , and N. Mersaditabari . 2013 Experimentally‐induced dissociation impairs visual memory. Conscious. Cogn. 22:1189–1194. 24021847 10.1016/j.concog.2013.07.007

[brb3474-bib-0010] Brewin, C. R. , B. Y. T. Ma , and J. Colson . 2013 Effects of experimentally induced dissociation on attention and memory. Conscious. Cogn. 22:315–323. 22959888 10.1016/j.concog.2012.08.005

[brb3474-bib-0011] Brown, A. D. , J. C. Root , T. A. Romano , L. J. Chang , R. A. Bryant , and W. Hirst . 2013 Overgeneralized autobiographical memory and future thinking in combat veterans with posttraumatic stress disorder. J. Behav. Ther. Exp. Psychiatry 44:129–134. 22200095 10.1016/j.jbtep.2011.11.004

[brb3474-bib-0012] Chae, Y. , G. Goodman , and R. Edelstein . 2011 Autobiographical memory development from an attachment perspective: the special role of negative events. Adv. Child Dev. Behav. 40:1–49. 21887958 10.1016/b978-0-12-386491-8.00001-3

[brb3474-bib-0013] Cohen, B. E. , T. C. Neylan , K. Yaffe , K. W. Samuelson , Y. Li , and D. E. Barnes . 2013 Posttraumatic stress disorder and cognitive function: findings from the mind your heart study. J. Clin. Psychiatry 74:1063–1070. 24330891 10.4088/JCP.12m08291

[brb3474-bib-0014] Conners, C. . 2000 Conners' continuous performance test II: computer program for Windows technical guide and software manual. Multi‐Health Systems, North Tonawanda, NY.

[brb3474-bib-0015] Conway, M. A. , and C. W. Pleydell‐Pearce . 2000 The construction of autobiographical memories in the self‐memory system. Psychol. Rev. 107:261–288. 10789197 10.1037/0033-295x.107.2.261

[brb3474-bib-0016] Dalgleish, T. , J. M. G. Williams , A.‐M. J. Golden , N. Perkins , L. F. Barrett , P. J. Barnard , et al. 2007 Reduced specificity of autobiographical memory and depression: the role of executive control. J. Exp. Psychol. Gen. 136:23–42. 17324083 10.1037/0096-3445.136.1.23 PMC2225543

[brb3474-bib-0017] D'Elia, L. , P. Satz , C. Uchiyama , and T. White . 1996 Color trails test. Psychological Assessment Resources, Odessa, FL.

[brb3474-bib-0018] Delis, D. C. , J. H. Kramer , E. Kaplan , and B. A. Ober . 2000 California Verbal Learning Test – second edition. Adult version. Manual. Psychological Corporation, San Antonio, TX.

[brb3474-bib-0019] Duggan, C. , P. Sham , C. Minne , A. Lee , and R. Murray . 1998 Quality of parenting and vulnerability to depression: results from a family study. Psychol. Med. 28:185–191. 9483695 10.1017/s0033291797006016

[brb3474-bib-0020] First, M. B. , R. L. Spitzer , M. Gibbon , and J. B. W. Williams . 1997 Structured Clinical Interview for DSM‐IV Axis I Disorders, Clinician Version (SCID‐CV). for DSMIV.

[brb3474-bib-0021] Fivush, R. , and A. Vasudeva . 2002 Reminiscing and relating: correlations among maternal reminscing style, attachment and emotional warmth. J. Cogn. Dev. 3:73–90.

[brb3474-bib-0022] Fivush, R. , C. A. Haden , and E. Reese . 2006 Elaborating on elaborations: role of maternal reminiscing style in cognitive and socioemotional development. Child Dev. 77:1568–1588. 17107447 10.1111/j.1467-8624.2006.00960.x

[brb3474-bib-0023] Gałecki, P. , M. Talarowska , D. Moczulski , K. Bobińska , K. Opuchlik , E. Gałecka , et al. 2013 Working memory impairment as a common component in recurrent depressive disorder and certain somatic diseases. Activitas Nervosa Superior Rediviva 55:47–56. 23922050

[brb3474-bib-0024] Gladsjo, J. A. , C. C. Schuman , J. D. Evans , G. M. Peavy , S. W. Miller , and R. K. Heaton . 1999 Norms for letter and category fluency: demographic corrections for age, education, and ethnicity. Assessment 6:147–178. 10335019 10.1177/107319119900600204

[brb3474-bib-0025] Gotlib, I. H. , J. H. Mount , N. I. Cordy , and V. E. Whiffen . 1988 Depression and perceptions of early parenting: a longitudinal investigation. Br. J. Psychiatry 152:24–27. 3167341 10.1192/bjp.152.1.24

[brb3474-bib-0026] Haaland, V. , and N. I. Landrø . 2009 Pathological dissociation and neuropsychological functioning in borderline personality disorder. Acta Psychiatr. Scand. 119:383–392. 19120046 10.1111/j.1600-0447.2008.01323.x

[brb3474-bib-0027] Hach, S. , L. J. Tippett , and D. R. Addis . 2014 Neural changes associated with the generation of specific past and future events in depression. Neuropsychologia 65:41–55. 25447064 10.1016/j.neuropsychologia.2014.10.003

[brb3474-bib-0028] Hamilton, M. 1960 A rating scale for depression. J. Neurol. Neurosurg. Psychiatry 23:56–62. 14399272 10.1136/jnnp.23.1.56 PMC495331

[brb3474-bib-0029] Harley, K. , and E. Reese . 1999 Origins of autobiographical memory. Dev. Psychol. 35:1338–1348. 10493658 10.1037//0012-1649.35.5.1338

[brb3474-bib-0030] Heaton, R. . 2003 Wisconsin card sorting test computer version 4 – research edition (WCST:CV4). Psychological Assessment Resources, Odessa, FL.

[brb3474-bib-0031] Holmes, E. A. , T. J. Lang , M. L. Moulds , and A. M. Steele . 2008 Prospective and positive mental imagery deficits in dysphoria. Behav. Res. Ther. 46:976–981. 18538304 10.1016/j.brat.2008.04.009

[brb3474-bib-0032] Joëls, M. 2011 Impact of glucocorticoids on brain function: relevance for mood disorders. Psychoneuroendocrinology 36:406–414. 20382481 10.1016/j.psyneuen.2010.03.004

[brb3474-bib-0033] Kensinger, E. A. 2009 Remembering the details: effects of emotion. Emot. Rev. 1:99–113. 19421427 10.1177/1754073908100432 PMC2676782

[brb3474-bib-0034] King, M. J. , A. G. MacDougall , S. M. Ferris , B. Levine , G. M. MacQueen , and M. C. McKinnon . 2010 A review of factors that moderate autobiographical memory performance in patients with major depressive disorder. J. Clin. Exp. Neuropsychol. 32:1122–1144. 20544462 10.1080/13803391003781874

[brb3474-bib-0035] King, M. J. , A. G. MacDougall , S. Ferris , K. A. Herdman , and M. C. McKinnon . 2011a Episodic simulation of future events is impaired in patients with major depressive disorder. Psychiatry Res. 187:465–467. 21377216 10.1016/j.psychres.2011.02.002

[brb3474-bib-0036] King, M. J. , L. A. Williams , A. G. MacDougall , S. Ferris , J. R. V. Smith , N. Ziolkowski , et al. 2011b Patients with bipolar disorder show a selective deficit in the episodic simulation of future events. Conscious. Cogn. 20:1801–1807. 21664146 10.1016/j.concog.2011.05.005

[brb3474-bib-0037] Kleim, B. , B. Graham , S. Fihosy , R. Stott , and A. Ehlers . 2014 Reduced specificity in episodic future thinking in posttraumatic stress disorder. Clin. Psychol. Sci. 2:165–173. 24926418 10.1177/2167702613495199 PMC4051242

[brb3474-bib-0038] Kosnes, L. , R. Whelan , A. O'Donovan , and L. A. McHugh . 2013 Implicit measurement of positive and negative future thinking as a predictor of depressive symptoms and hopelessness. Conscious. Cogn. 22:898–912. 23810864 10.1016/j.concog.2013.06.001

[brb3474-bib-0039] Krause‐Utz, A. , N. Y. L. Oei , I. Niedtfeld , M. Bohus , P. Spinhoven , C. Schmahl , et al. 2012 Influence of emotional distraction on working memory performance in borderline personality disorder. Psychol. Med. 42:2181–2192. 22397907 10.1017/S0033291712000153

[brb3474-bib-0040] Levine, B. , E. Svoboda , J. F. Hay , G. Winocur , and M. Moscovitch . 2002 Aging and autobiographical memory: dissociating episodic from semantic retrieval. Psychol. Aging 17:677–689. 12507363

[brb3474-bib-0041] MacLeod, A. , and A. Byrne . 1997 Anxiety, depression, and the anticipation of future positive and negative experiences. J. Abnorm. Psychol. 105:286–289. 10.1037//0021-843x.105.2.286 8723011

[brb3474-bib-0042] McDermott, L. M. , and K. P. Ebmeier . 2009 A meta‐analysis of depression severity and cognitive function. J. Affect. Disord. 119:1–8. 19428120 10.1016/j.jad.2009.04.022

[brb3474-bib-0043] McKinnon, M. C. , E. I. Nica , P. Sengdy , N. Kovacevic , M. Moscovitch , M. Freedman , et al. 2008 Autobiographical memory and patterns of brain atrophy in frontotemporal lobar degeneration. J. Cogn. Neurosci. 20:1839–1853. 18370601 10.1162/jocn.2008.20126 PMC6553881

[brb3474-bib-0044] McKinnon, M. C. , D. J. Palombo , A. Nazarov , N. Kumar , W. Khuu , and B. Levine . 2014 Threat of death and autobiographical memory: a study of passengers from flight AT236. Clin. Psychol. Sci. 3:487–502. 26167422 10.1177/2167702614542280 PMC4495962

[brb3474-bib-0045] van der Meere, J. , N. Börger , and T. van Os . 2007 Sustained attention in major unipolar depression. Percept. Mot. Skills 104(3 Pt 2):1350–1354. 17879669 10.2466/pms.104.4.1350-1354

[brb3474-bib-0046] Molina‐Serrano, A. , S. Linotte , M. Amat , D. Souery , and M. Barreto . 2008 Dissociation in major depressive disorder: a pilot study. J. Trauma Dissociation 9:411–421. 19042786 10.1080/15299730802139311

[brb3474-bib-0047] Moore, S. A. , and L. A. Zoellner . 2007 Overgeneral autobiographical memory and traumatic events: an evaluative review. Psychol. Bull. 133:419–437. 17469985 10.1037/0033-2909.133.3.419 PMC2665927

[brb3474-bib-0048] Morina, N. , C. Deeprose , C. Pusowski , M. Schmid , and E. A. Holmes . 2011 Prospective mental imagery in patients with major depressive disorder or anxiety disorders. J. Anxiety Disord. 25:1032–1037. 21783339 10.1016/j.janxdis.2011.06.012 PMC3389342

[brb3474-bib-0050] Nelson, K. , and R. Fivush . 2004 The emergence of autobiographical memory: a social cultural developmental theory. Psychol. Rev. 111:486–511. 15065919 10.1037/0033-295X.111.2.486

[brb3474-bib-0051] Noguchi, K. , Y. R. Gel , E. Brunner , and F. Konietschke . 2012 nparLD: an R software package for the nonparametric analysis of longitudinal data in factorial experiments. J. Stat. Softw. 50:1–23.25317082

[brb3474-bib-0052] Paivio, A. , J. C. Yuille , and S. A. Madigan . 1968 Concreteness, imagery, and meaningfulness values for 925 nouns. J. Exp. Psychol. 76(Suppl):1–25. 5672258 10.1037/h0025327

[brb3474-bib-0053] Parker, G. , H. Tupling , and L. B. Brown . 1979 A parental bonding instrument. Br. J. Med. Psychol. 52:1–10.

[brb3474-bib-0054] Peterson, C. , and D. T. K. Nguyen . 2010 Parent‐child relationship quality and infantile amnesia in adults. Br. J. Psychol. 101:719–737. 20100396 10.1348/000712609X482948

[brb3474-bib-0055] Polak, A. R. , A. B. Witteveen , J. B. Reitsma , and M. Olff . 2012 The role of executive function in posttraumatic stress disorder: a systematic review. J. Affect. Disord. 141:11–21. 22310036 10.1016/j.jad.2012.01.001

[brb3474-bib-0056] Raes, F. , D. Hermans , J. M. G. Williams , K. Demyttenaere , B. Sabbe , G. Pieters , et al. 2005 Reduced specificity of autobiographical memory: a mediator between rumination and ineffective social problem‐solving in major depression? J. Affect. Disord. 87:331–335. 15979154 10.1016/j.jad.2005.05.004

[brb3474-bib-0057] Reese, E. , and R. Fivush . 1993 Parental styles of talking about the past. Dev. Psychol. 29:596–606.

[brb3474-bib-0058] Roca, V. , J. Hart , T. Kimbrell , and T. Freeman . 2006 Cognitive function and chronic posttraumatic stress study. J. Neuropsychiatry Clin. Neurosci. 18:226–230. 16720800 10.1176/jnp.2006.18.2.226

[brb3474-bib-0059] Schacter, D. L. , and D. R. Addis . 2007 The cognitive neuroscience of constructive memory: remembering the past and imagining the future. Philos. Trans. R. Soc. Lond. B Biol. Sci. 362:773–786. 17395575 10.1098/rstb.2007.2087 PMC2429996

[brb3474-bib-0060] Selcuk, E. , V. Zayas , G. Günaydin , C. Hazan , and E. Kross . 2012 Mental representations of attachment figures facilitate recovery following upsetting autobiographical memory recall. J. Pers. Soc. Psychol. 103:362–378. 22486677 10.1037/a0028125

[brb3474-bib-0061] Snyder, H. R. 2012 Major depressive disorder is associated with broad impairments on neuropsychological measures of executive function: a meta‐analysis and review. Psychol. Bull. 139:81–132. 22642228 10.1037/a0028727 PMC3436964

[brb3474-bib-0062] Spinhoven, P. , C. L. H. Bockting , A. H. Schene , M. W. J. Koeter , E. M. Wekking , and J. M. G. Williams . 2006 Autobiographical memory in the euthymic phase of recurrent depression. J. Abnorm. Psychol. 115:590–600. 16866599 10.1037/0021-843X.115.3.590

[brb3474-bib-0063] Spinhoven, P. , B. W. Penninx , A. M. van Hemert , M. de Rooij , and B. M. Elzinga . 2014 Comorbidity of PTSD in anxiety and depressive disorders: prevalence and shared risk factors. Child Abuse Negl. 38:1320–1330. 24629482 10.1016/j.chiabu.2014.01.017

[brb3474-bib-0064] Spreng, R. N. , and B. Levine . 2006 The temporal distribution of past and future autobiographical events across the lifespan. Mem. Cognit. 34:1644–1651. 10.3758/bf03195927 PMC1942074 17489291

[brb3474-bib-0065] Sumner, J. A. , S. Mineka , E. K. Adam , M. G. Craske , S. Vrshek‐Schallhorn , K. Wolitzky‐Taylor , et al. 2014 Testing the CaR‐FA‐X model: investigating the mechanisms underlying reduced autobiographical memory specificity in individuals with and without a history of depression. J. Abnorm. Psychol. 123:471–486. 24978693 10.1037/a0037271 PMC4122646

[brb3474-bib-0066] Talarowska, M. , A. Florkowski , K. Zboralski , D. Berent , P. Wierzbiński , and P. Gałecki . 2010 Auditory‐verbal declarative and operating memory among patients suffering from depressive disorders – preliminary study. Adv. Med. Sci. 55:317–327. 21163755 10.2478/v10039-010-0053-0

[brb3474-bib-0067] Tulving, E. 2002 Episodic memory: from mind to brain. Annu. Rev. Psychol. 53:1–25. 11752477 10.1146/annurev.psych.53.100901.135114

[brb3474-bib-0068] Vasterling, J. J. , L. M. Duke , K. Brailey , J. I. Constans , A. N. Allain , and P. B. Sutker . 2002 Attention, learning, and memory performances and intellectual resources in Vietnam veterans: PTSD and no disorder comparisons. Neuropsychology 16:5–14. 11853357 10.1037//0894-4105.16.1.5

[brb3474-bib-0069] Wechsler, D. 1999 Wechsler Abbreviated Scale of Intelligence (WASI) manual. Psychological Corporation, San Antonio, TX.

[brb3474-bib-0070] Williams, J. M. G. , T. Barnhofer , C. Crane , D. Herman , F. Raes , E. Watkins , et al. 2007 Autobiographical memory specificity and emotional disorder. Psychol. Bull. 133:122–148. 17201573 10.1037/0033-2909.133.1.122 PMC2834574

[brb3474-bib-0071] Yehuda, R. , J. A. Golier , S. L. Halligan , and P. D. Harvey . 2004 Learning and memory in Holocaust survivors with posttraumatic stress disorder. Biol. Psychiatry 55:291–295. 14744471 10.1016/s0006-3223(03)00641-3

[brb3474-bib-0072] Žikić, O. , S. Ćirić , and M. Mitković . 2009 Depressive phenomenology in regard to depersonalization level. Psychiatr. Danub. 21:320–326. 19794348

